# EHMTI-0353. MR tractography in short lasting unilateral neuralgiform headache attacks with conjunctival injection and tearing (SUNCT) patients: case reports

**DOI:** 10.1186/1129-2377-15-S1-I10

**Published:** 2014-09-18

**Authors:** O Coskun, M Ucar, F Yildirim, R Cetinkaya, H Bolay

**Affiliations:** 1 Neurology, Gazi University School of Medicine, Ankara, Turkey; 2 Radiology, Gazi University School of Medicine, Ankara, Turkey

## 

Short-lasting unilateral neuralgiform headache attacks with conjunctival injection and tearing (SUNCT) is trigeminal autonomic cephalalgias which is characterized by repetitive short lasting, severe attacks. Headache attacks are distribution of the ophtalmic and maxillary trigeminal divisions and associated with ipsilateral autonomic phenomena .A growing body of literature has focused on brain magnetic resonance imaging (MRI) evidence of neurovascular compression in these syndromes. There is some evidence supporting microvascular decompression of the trigeminal nerve in selected patients who have medically refractory SUNCT and a demonstrable ipsilateral aberrant vessel on magnetic resonance imaging (MRI). Here, we describe two cases concerning a 52-year-adult and a 69-year-elderly with short-lasting, recurrent headache combined with cranial autonomic features. Pain was described as excruciating, and was non-responsive to most traditional analgesic drugs. We report two patients of SUNCT syndrome with MRI cisternography and tractography findings of neurovascular compression. We performed MRI cisternography and tractography for delineates structural changes in the trigeminal nerve. Pain was completely relieved after surgery that microvascular decompression.

We suggest that SUNCT patients with brain MRI should always be performed with a dedicated view to exclude neurovascular compression. In this case reports we performed MRI tractography delineates structural changes in the trigeminal nerve for SUNCT. MRI tractography is the first reports for SUNCT patients in the literature.

